# Fruit quality and volatile constituents of a new very early-ripening pummelo (*Citrus maxima*) cultivar ‘Liuyuezao’

**DOI:** 10.3389/fpls.2022.1089009

**Published:** 2023-01-09

**Authors:** Tengfei Pan, Lingchao Kong, Xinxin Zhang, Yanhui Wang, Jinyu Zhou, Zhijun Fu, Heli Pan, Wenqin She, Yuan Yu

**Affiliations:** ^1^ College of Horticulture, Fujian Agriculture and Forestry University, Fuzhou, Fujian, China; ^2^ FAFU-UCR Joint Center for Horticultural Biology and Metabolomics, Haixia Institute of Science and Technology, Fujian Agriculture and Forestry University, Fuzhou, Fujian, China

**Keywords:** citrus, new cultivar, breeding, flavor, phytochemicals

## Abstract

‘Liuyuezao’ (LYZ) pummelo (*Citrus maxima*) originated from a spontaneous bud sport on a ‘Guanxi’ (GXB) pummelo tree and was released as a new very early-season cultivar. The objective of this study was to present the sensory and nutritional profiles of LYZ fruits, and compare it with other major commercialized pummelo cultivars including GXB, ‘Sanhong’ (SH) and ‘Hongrou’ (HR). LYZ had higher contents of organic acids (12.01 mg/g), phenols (669.01 mg/L), vitamin C (75.73 mg/100 mL) and stronger antioxidant capacity (77.65 mg/100 mL) but lower levels of soluble sugars (62.85 mg/g), carotenoids (0.25 mg/L) and flavonoids (46.3 mg/L) when compared to the other pummelos. Moreover, a smaller number (49) and much less content (7.63) of fruit volatiles were detected in LYZ than them in GXB, SH and HR. The relatively high levels of fructose (20.6 mg/g) and organic acids and low levels of volatile compounds in LYZ mainly contributed to its sweet and mildly sour taste and moderate aroma of pummelo note. LYZ is presented as an alternative pummelo cultivar with the potential for commercialization.

## 1 Introduction

Citrus, one of the largest internationally traded agricultural products, is popular with consumers around the world for its bright color, savory taste and strong aroma. It is well known that pummelo is monoembryonic, and it has formed a highly heterozygous genetic composition with a high bud mutation rate and easy hybridization with other citrus species during the long period of sexual reproduction. Fruits including citrus are among the healthiest foods in horticultural crops, they ‘re delicious, nutritious, and provide a number of impressive health benefits (Bolat et al., 2014; Ersoy et al., 2018; Gecer et al., 2020; Grygorieva et al., 2021; Jurić et al., 2021) Citrus flavedo can be used to refine essential oils for its large amount of volatile compounds (Gonzalez-Mas et al., 2019). For citrus pulp, several studies have mentioned its medical value. For example, Kelebek (2010) pointed out that the pummelo pulp contained high levels of vitamin C, phenols and microelements which are beneficial to the treatment of obesity, diabetes, cancer and cardiovascular diseases. Flavonoids, as essential substances for the human body due to their high medicinal value and antioxidant properties, are abundant in pummelo pulp (Wang et al., 2019).

Taste is of paramount importance for citrus fruits, and soluble sugars and organic acids are the most important factors affecting taste. The soluble sugar content is a crucial factor affecting the fruit flavor due to the high accuracy and wide adaptability in reflecting fruit sweetness. The main components of soluble sugars in pummelo fruit are glucose, fructose and sucrose, among which the sweetness ratio was 1.7: 1: 0.75 (Mao et al., 2019). The sweetness of pummelo fruit is not only related to the total content of soluble sugars but also related to the sugar composition. The organic acids in pummelo fruit are largely stored in the pulp cytoplasmic vacuoles which mainly include citric acid and malic acid, and citric acid accounted for 39.1% - 63.55% of the total acids (Zheng et al., 2016).

Flavor incorporates human evaluation of taste and aroma, and the comprehensive feeling of good flavor can form a cheerful feeling in the brain (Goff and Klee, 2006; Tournier et al., 2009; Tietel et al., 2011; Thomas-Danguin et al., 2016). As a result, the aroma becomes increasingly important to the fruit. Prevalent volatile compounds in pummelo flavedo are mainly terpenes and alcohols, and limonene and β-myrcene are the top 2 volatile compounds among pummelo flavedo volatiles (Gonzalez-Mas et al., 2019). The common volatile compounds in pummelo pulp are primarily alcohols, aldehydes and terpenes, and the number and total content of volatiles are less in pulp than in flavedo.

‘Liuyuezao’ (LYZ) is a new very early-season pummelo cultivar developed from a spontaneous bud sport on a ‘Guanxi’ (GXB) pummelo tree (Pan et al., 2021). LYZ pummelo ripens in late July to early August in Fujian, and enters the market about 45 days earlier than the other major cultivars including GXB, ‘Hongrou’ (HR) and ‘Sanhong’ (SH). In this study, GXB, HR and SH were used to compare with LYZ in the fruit characteristics as well as sensory and nutritional profiles.

## 2 Materials and methods

### 2.1 Plant materials

LYZ, GXB, HR and SH were all produced in Pinghe, Fujian, China. The pummelo trees were grafted on sour pummelo rootstock in 2009 and cultivated in a pummelo orchard under normal pest control and fertilizer management. 15 mature and disease-free fruits were randomly sampled from three trees and gathered as one biological replicate, and three biological replicates were prepared. The pulp and juice samples were immediately collected and rapidly frozen in liquid nitrogen and stored at -80°C for later use. Each measurement for fruit sensory and nutritional traits included three technical replications.

### 2.2 Chemicals and reagents

3-hexanone (chromatographic grade, Sigma-Aldrich); C8-C20 alkane mixture (Sigma-Aldrich); n-hexane (chromatographic grade, Ron); chloroform (AR, Sinopharm); Acetone (AR, Sinopharm); Folin-Ciocalteu (AR, Sinopharm); Gallic acid (AR, Sinopharm); Ascorbic acid (AR, Sinopharm); 2,2-diphenyl-1-picrylhydrazyl (AR, Sinopharm); 2,6-dichlorophenolindophenol (AR, Sinopharm); (+)-Catechin (AR, Sinopharm). 30 authentic volatile compound standards (Sigma-Aldrich).

### 2.3 Fruit sensory quality

#### 2.3.1 Soluble sugars

Soluble sugars were measured through a high-performance liquid chromatography (HPLC). 2 g pulp samples (ground in liquid nitrogen) were mixed with 10 mL of 95% methanol. The supernatant was collected after sonication for 30 min at 40°C and centrifugation for 10 min at 10,000 g. The clear fluid was filtered and collected. Soluble sugars were divided by an Ellistat Supersil NH_2_ column (4.6 mm × 250 mm, 5 μm particle size) (Waters Inc, Zellik, Belgium) at 40°C. The mobile phase was comprised of solvent A (82% acetonitrile) and solvent B (18% ultrapure water). Soluble sugars (mg/g) were calculated using the calibration curves of the corresponding standards.

#### 2.3.2 Organic acids

Organic acids were determined by the modified method (Nour et al., 2010). Briefly, 2 g samples (ground in liquid nitrogen) were mixed with 10 ml of 95% methanol. The supernatant was diluted 25 times and filtered after centrifugation for 15 min at 2500 g. Organic acids were evaluated by an ultra-performance liquid chromatography (UPLC) coupled with an Acquity HSS T3 chromatography column (1.8 μm particle size, 2.1 mm × 100 mm). The mobile phase was (NH4)_2_HPO_4_ (50 mmol/L, pH 2.7) with a 0.2 mL/min flow rate. Organic acids (mg/g) were quantified by the calibration curves of the corresponding standards.

#### 2.3.3 Lignin content

Lignin content was determined using the method described by Fukushima and Kerley (2011). Briefly, 50 mg of dried pulp powder was placed in a 10 mL centrifuge tube, washed respectively three times with 5 mL of deionized water, 2 mL of 95% ethanol, 2 mL of acetone, and 2 mL of ether (centrifuged at 8000 g for 2 min in the middle). The precipitate was dissolved in the mixture (1 mL of 2 mol/L NaOH, 0.1 mL 7.5 mol/L hydroxylamine hydrochloride) and diluted to 10 mL with acetic acid. The absorbance values were measured at 280 nm in comparison with the prepared blank. The results (%) were calculated based on the corresponding calibration curve.

### 2.4 Nutritional quality

#### 2.4.1 Total carotenoids

Total carotenoids in pummelo samples were determined using the method reported by Goldenberg et al. (2014). Briefly, 0.5 mL of juice and 1 mL of extraction fluid (hexane/acetone/ethanol = 2/1/1) were homogenized for 30 s, followed by centrifugation at 4730 g for 5 min at 5°C. Absorbance values of the chromogenic layer were determined at 450 nm using a Multiscan Spectrum microplate reader (Thermo Electron Corporation, S.A.), and total carotenoids (mg/L) were calculated according to the formula of β-carotene extinction coefficient E^1%^=2400.

#### 2.4.2 Total phenols and flavonoids

Phenols, flavonoids and antioxidant capacity of the pummelo samples were extracted according to the method reported by Goldenberg et al. (2014). The Folin-Ciocalteu method (Singleton et al., 1999) was used to determine the total phenols. Briefly, the reaction mixture consisted of 0.1 mL of juice methanol extract, 0.5 mL of double-distilled water, 0.1 mL of Folin-Ciocalteu reagent, and 1 mL Na_2_CO_3_ solution. The reaction mixture was reacted at room temperature for 90 min in dark and the absorbance values were measured at 750 nm in comparison with the prepared blank. Total phenols (mg/L) were expressed as gallic acid equivalents.

Total flavonoids were determined based on the method described by Shin et al. (2007). The reaction mixture consisted of 0.5 mL of methanolic juice extract and 0.12 mL of 5% NaNO_2_, followed by adding 0.12 mL of 10% Al(NO_3_)_3_ after 5 min, and the reaction was terminated by adding 1 mL of 1 mol/L NaOH solution after 6 min at room temperature. The content of flavonoids was measured by comparing the absorbance at 510 nm with the prepared blank, and the results (mg/L) were expressed as catechin equivalents.

#### 2.4.3 Antioxidant capacity

The antioxidant capacity of pummelo juice was evaluated according to the 2,2-diphenyl-1-picrylhydrazyl (DPPH) method (Sdiri et al., 2012). Briefly, 0.1 mL of methanolic extract of juice, 0.5 mL of double-distilled water, and 0.8 mL of DPPH solution (0.2 mmol/L) were mixed completely, followed by 30 min protection from light. The result was calculated using the absorbance at 517 nm by a Multiscan Spectrum microplate reader (Thermo Electron Corporation, S.A.), and the antioxidant capacity (mg/100 mL) was expressed as the ascorbic acid equivalents.

#### 2.4.4 Vitamin C

Vitamin C (ascorbic acid) content was determined by titration against 2,6-dichlorophenolindophenol (Hiromi et al., 1980). Ascorbic acid content (mg/100 mL) was presented as milligrams of ascorbic acid per 100 mL of juice.

### 2.5 Volatile determination

The separation and concentration of pummelo volatiles were performed by headspace solid phase microextraction (SPME) (Yu et al., 2018). 4 g of sample powder was mixed completely with 4 mL of saturated sodium chloride solution in a 20 mL headspace vial, and then 6 μL of 1000 ppm 3-hexanone was added as an internal standard. The headspace vials were sealed with polytetrafluoroethylene septum (Gerstel Inc., Linthicum, MD, USA) and stored at -20°C. To fully extract volatiles, the SPME fiber (1 cm, 50/30 μm, Divinylbenzene/Carboxen/Polydimethylsiloxane) was exposed to the headspace vial for 60 min at 40°C.

Volatile compounds were dissociated and identified by the gas chromatography (7890B, Agilent Technologies, Santa Clara) - mass spectrometer (LECO, Saint Joseph) (GC-MS) equipped with a DB-5MS column (30 m × 0.25 μm × 0.25 μm, Rxi-5 Sil MS, Restek, Bellefonte). The column oven temperature program was held from 40°C to 230°C at 3° C/min and rose to 260°C at 15° C/min, and remained for 5 min. Volatile data from 30-500 m/z was collected by 70 eV electron ionization (EI) with a set temperature of 250°C. The retention indices (RI) were calculated using a C8-C20 mixed standard (40 mg/L, final concentration was 10 mg/L).

Volatile compounds were qualified by comparing the collected chromatographic peaks with mass spectral databases such as Wiley HPCH 2205.L, NIST05.L and ADAMS.L (Adams, 2007). The RIs of the collected compounds were compared with the reported RI value to further confirmation. The relative content of aroma volatiles was quantified based on its peak area exceeding the internal standard.

### 2.6 Statistical analysis

Significant differences were calculated using one-way ANOVA based on Tukey’s multiple range test (p ≤ 0.05) by SPSS statistical software. Principal component analysis (PCA) was performed using JMP 13.0 software. Hierarchical cluster analysis (HCA) based on Ward’s method was carried out by the R software. The Pearson correlation analysis was completed by Origin 2021 software.

## 3 Results and discussion

### 3.1 LYZ fruit characteristics

**Figure 1:**
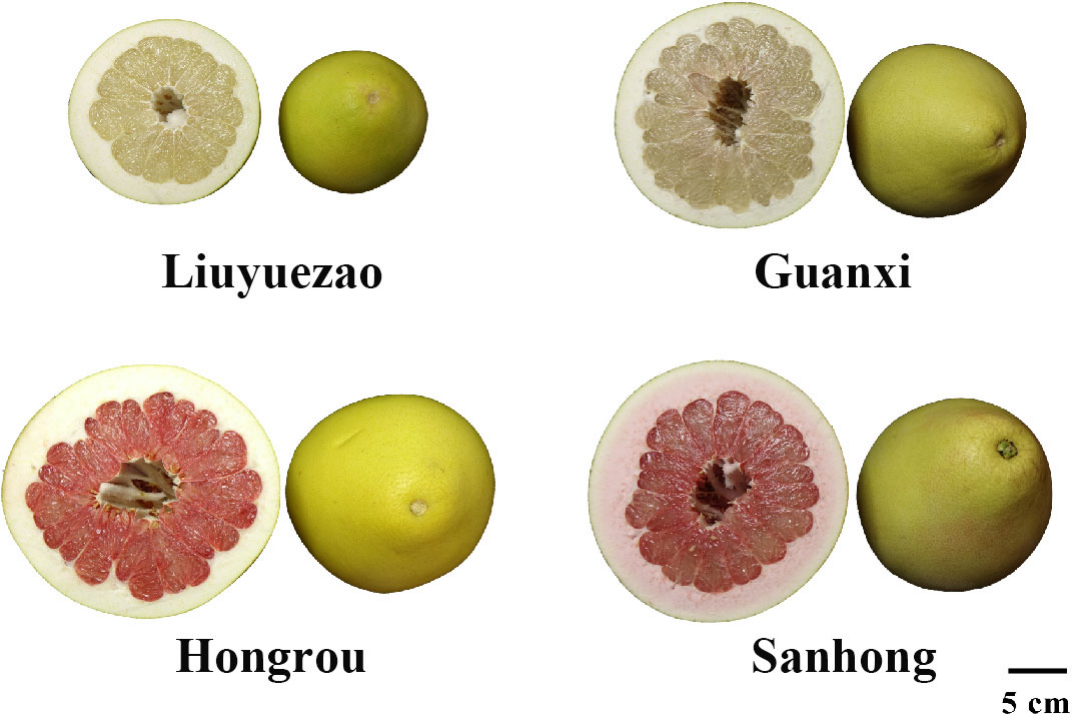
Fruits of the four pummelo cultivars.

Consumers pay great importance to the fruit appearance. The fruit characteristics including fruit weight, diameter, height, shape index (height/diameter ratio), pulp weight, eatable rate and flavedo thickness were measured among the four pummelo cultivars. The LYZ fruits developed a light yellow and smooth rind and a pale yellow flesh (**Figure 1**). Compared to HR, SH and GXB, LYZ displayed a comparable fruit diameter (14.01 cm), fruit weight (1243.27 g) and eatable rate (0.71), but a significantly higher shape index (1.06) (**Table 1**). Hierarchical cluster analysis (HCA) based on the fruit traits could not separate LYZ from the other three cultivars, which indicated that LYZ fruits were not greatly special in fruit characteristics (**Figure S1**).

**Table 1:** Fruit quality traits of the four pummelo cultivars.

Trait	Hongrou	Sanhong	Guanxi	Liuyuezao
Fruit weight (g)	1109.99 ± 184.26 ^c^	1442.07 ± 113.41 ^a^	1268.90 ± 136.48 ^b^	1243.27 ± 138.33 ^bc^
Fruit diameter (cm)	13.64 ± 0.47 ^b^	16.73 ± 1.39 ^a^	14.28 ± 0.69 ^b^	14.01 ± 1.03 ^b^
Fruit height (cm)	13.60 ± 0.80 ^c^	16.99 ± 1.30 ^a^	14.33 ± 0.95 ^bc^	14.87 ± 1.14 ^b^
Shape index	1.00 ± 0.05 ^b^	1.02 ± 0.05 ^b^	1.00 ± 0.05 ^b^	1.06 ± 0.05 ^a^
Pulp weight (g)	869.67 ± 161.31 ^b^	1009.34 ± 82.04 ^a^	981.68 ± 113.43 ^a^	880.59 ± 118.42 ^b^
Eatable rate (%)	0.79 ± 0.10 ^a^	0.70 ± 0.01 ^b^	0.77 ± 0.04 ^a^	0.71 ± 0.03 ^b^
Flavedo thickness (cm)	1.35 ± 0.25 ^a^	1.42 ± 0.10 ^a^	1.52 ± 0.15 ^a^	1.10 ± 0.11 ^b^
Citric a cid (mg/g)	6.60 ± 0.48 ^b^	5.72 ± 0.25 ^b^	6.58 ± 0.51 ^b^	9.08 ± 1.40 ^a^
Malic acid (mg/g)	0.38 ± 0.05 ^b^	0.34 ± 0.02 ^b^	0.30 ± 0.08 ^b^	1.42 ± 0.57 ^a^
Titratable acids (mg/g)	8.56 ± 0.59 ^b^	6.96 ± 0.29 ^c^	7.90 ± 0.61 ^bc^	12.01 ± 0.65 ^a^
Fructose (mg/g)	19.80 ± 0.36 ^a^	18.70 ± 0.86 ^b^	18.17 ± 0.13 ^b^	20.60 ± 0.54 ^a^
Glucose (mg/g)	19.31 ± 0.91 ^ab^	18.33 ± 0.74 ^b^	17.91 ± 0.26 ^b^	21.01 ± 0.60 ^a^
Sucrose (mg/g)	55.28 ± 2.36 ^a^	40.92 ± 1.10 ^b^	39.49 ± 2.68 ^b^	21.16 ± 1.27 ^c^
Soluble sugars (mg/g)	94.47 ± 3.37 ^a^	77.96 ± 0.66 ^b^	75.57 ± 2.29 ^b^	62.85 ± 2.38 ^c^
Fructose/sucrose	0.36 ± 0.02 ^c^	0.46 ± 0.03 ^b^	0.46 ± 0.04 ^b^	0.98 ± 0.04 ^a^
Soluble sugars/organic acids	11.04 ± 0.27 ^a^	11.20 ± 0.27 ^a^	9.57 ± 0.18 ^b^	5.25 ± 0.07 ^c^
Lignin (%)	2.90 ± 0.30 ^a^	1.52 ± 0.21 ^bc^	1.87 ± 0.31 ^b^	1.28 ± 0.06 ^c^
Carotenoids (mg/L)	4.35 ± 1.14 ^b^	6.44 ± 0.26 ^a^	0.44 ± 0.27 ^c^	0.25 ± 0.03 ^c^
Flavonoids (mg/L)	53.06 ± 3.69 ^a^	47.32 ± 4.72 ^a^	47.48 ± 1.66 ^a^	46.30 ± 2.06 ^a^
Antioxidant capacity (mg/100mL)	39.89 ± 2.68 ^b^	29.11 ± 3.02 ^c^	41.15 ± 3.34 ^b^	77.65 ± 1.94 ^a^
Phenols (mg/L)	372.51 ± 21.75 ^b^	306.47 ± 7.89 ^b^	298.5 ± 15.14 ^b^	669.01 ± 39.55 ^a^
Vitamin C (mg/100mL)	41.32 ± 2.60 ^b^	37.15 ± 1.30 ^b^	42.01 ± 2.60 ^b^	75.73 ± 2.10 ^a^

All the trees were grafted on sour pummelo rootstock in 2009. All values are mean ± SD of three biological replicates (15 fruits each) per cultivar; different letters in the same row indicate significant differences according to Tukey’s honestly significant difference test at P < 0.05.

### 3.2 LYZ fruit sensory and nutritional quality

To better describe the fruit quality of LYZ, the sensory and nutritional quality were analyzed in this study. The sensory quality included soluble sugars, organic acids and lignin content, and the nutritional quality contained carotenoids, phenols, flavonoids, vitamin C and antioxidant capacity.

#### 3.2.1 Soluble sugars

Soluble sugars were important factors in determining the sweetness of pummelo fruit (Hussain et al., 2020). The soluble sugar content of LYZ fruit was 62.85 mg/g, significantly lower than HR (94.47 mg/g), SH (77.96 mg/g) and GXB (75.57 mg/g) (**Table 1**). Fructose, sucrose and glucose were the main soluble sugars in citrus fruit. The content of fructose and glucose in LYZ fruit were 20.60 mg/g and 21.01 mg/g, respectively, both higher than the other three cultivars. However, the sucrose content in LYZ was 21.16 mg/g, approximately twice less than HR (55.28 mg/g), SH (40.92 mg/g) and GXB (39.49 mg/g) (**Table 1**).

It was notable that the fructose/sucrose ratio in LYZ was 0.98, much higher than the other three varieties (0.36-0.46) (**Table 1**). The study on ‘Huangjin’, ‘Sanhong’ and ‘Dongshizao’ pummelos showed that the content of sucrose was much higher than fructose and glucose (Zhu et al., 2020), which was different from the results in LYZ fruit. The content of fructose, sucrose and glucose was nearly equal in sweet orange fruit (Yu et al., 2012), and a similar sugar composition was found in LYZ. In addition, Kelebek and Selli (2011) also found that the orange fruit contained almost equal content of fructose and sucrose. Furthermore, the sweetness of fructose is about 1.5 times more than sucrose. As a result, the high fructose/sucrose ratio gave LYZ a unique sweetness even though LYZ fruit had a relatively low soluble sugar content.

#### 3.2.2 Organic acids

Free organic acids were crucial factors determining the flavor of pummelo fruit. In the present study, LYZ had the highest organic acid content (12.01 mg/g) among the four cultivars (**Table 1**). Most organic acids in citrus fruits were aliphatic carboxylic acids, such as citric acid and malic acid. Here, the content of citric acid and malic acid were 9.08 mg/g (75.60%) and 1.42 mg/g (11.82%), respectively, which indicated that LYZ was a citric acid fruit (**Table 1**). Citric acid was the most abundant and the biggest contributor to fruit acidity. The content of citric acid and malic acid in LYZ were about 1.48 times and 4.23 times as much as those in the other three pummelos. Additionally, citric acid and malic acid are different in acidity taste. Citric acid is a pleasant sour agent, while malic acid is bitter and the irritation could be retained for a long time. The concentration of the two main acids made LYZ a specific sour taste. During fruit ripening, the organic acids decreased in citrus fruit (Chen et al., 2009). Therefore, we believed that the high acidity in LYZ fruit was caused by early maturity, which was also reported in the study between early maturity and common citrus (Li et al., 2016).

LYZ fruit had a relatively high acid content and a low sugar content. The taste and palatability of citrus fruit mainly depend on the total soluble sugars/total organic acids ratio, and the ratio is also a commercial harvest indicator (Gao et al., 2018). The soluble sugars/organic acids ratio in LYZ fruit was 5.25, which was greatly lower than that of the other pummelo cultivars (9.57-11.2) (**Table 1**). In conclusion, the combination of the unique sweetness and high acidity generated the sweet and mildly acid taste of LYZ fruit.

#### 3.2.3 Lignin content

The unpleasant texture was directly caused by the excessive accumulation of lignin in citrus fruits (Shi et al., 2020). In this study, the lignin content in LYZ fruit was 1.28%, which confirmed the rare juice sac granulation in LYZ (**Table 1**). A total of 702 differentially expressed genes (DEGs) related to lignin content were found in HR fruit including 24 transcription factors (TFs) through metabolomics and transcriptomic analysis (Shi et al., 2020). LYZ, with a low level of lignin, could be a novel experimental material for fruit granulation study. Zhou et al. (2021) reported a certain correlation between lignin and soluble sugars, and a similar result was identified in our study where the lignin content was significantly correlated with the soluble sugars (0.909) (**Figure S3**).

#### 3.2.4 Nutritional quality

Nutritional quality reflects the contribution of the fruit to human health and is an important indicator to evaluate the fruit. Nowadays, many studies have shown that the natural antioxidants in fruits play an important role in improving the antioxidant status of the body, reducing inflammation and uric acid levels and protecting the cardiovascular system (Escudero-Lopez et al., 2018). The antioxidant activity of LYZ fruit was 77.65 mg/100 mL, which was much higher than that of the other three cultivars (29.11-41.15 mg/100 mL) (**Table 1**). The high antioxidant capacity of LYZ fruit could increase the consumers’ attention. In addition, phenols and vitamin C content in LYZ fruit were 669.01 mg/L and 75.73 mg/L, respectively, almost twice higher than those in the other three pummelos (**Table 1**). However, few carotenoids were detected in LYZ fruit, which mainly explained the white flesh of LYZ fruit. (**Table 1**). Vitamin C in citrus pulp made an important contribution to hydrophilic antioxidant capacity, while carotenoids contributed significantly to lipophilic antioxidant capacity (Zacarias-Garcia et al., 2021). It was previously reported that vitamin C was the largest contributor to the antioxidant capacity of citrus fruit (Goldenberg et al., 2014). However, in this study, the antioxidant capacity was positively correlated with vitamin C (0.98) and phenolics (0.95) (**Figure S3**), indicating that the strong antioxidant capacity of LYZ pulp was mainly caused by the high content of phenols and vitamin C.

**Figure 2:**
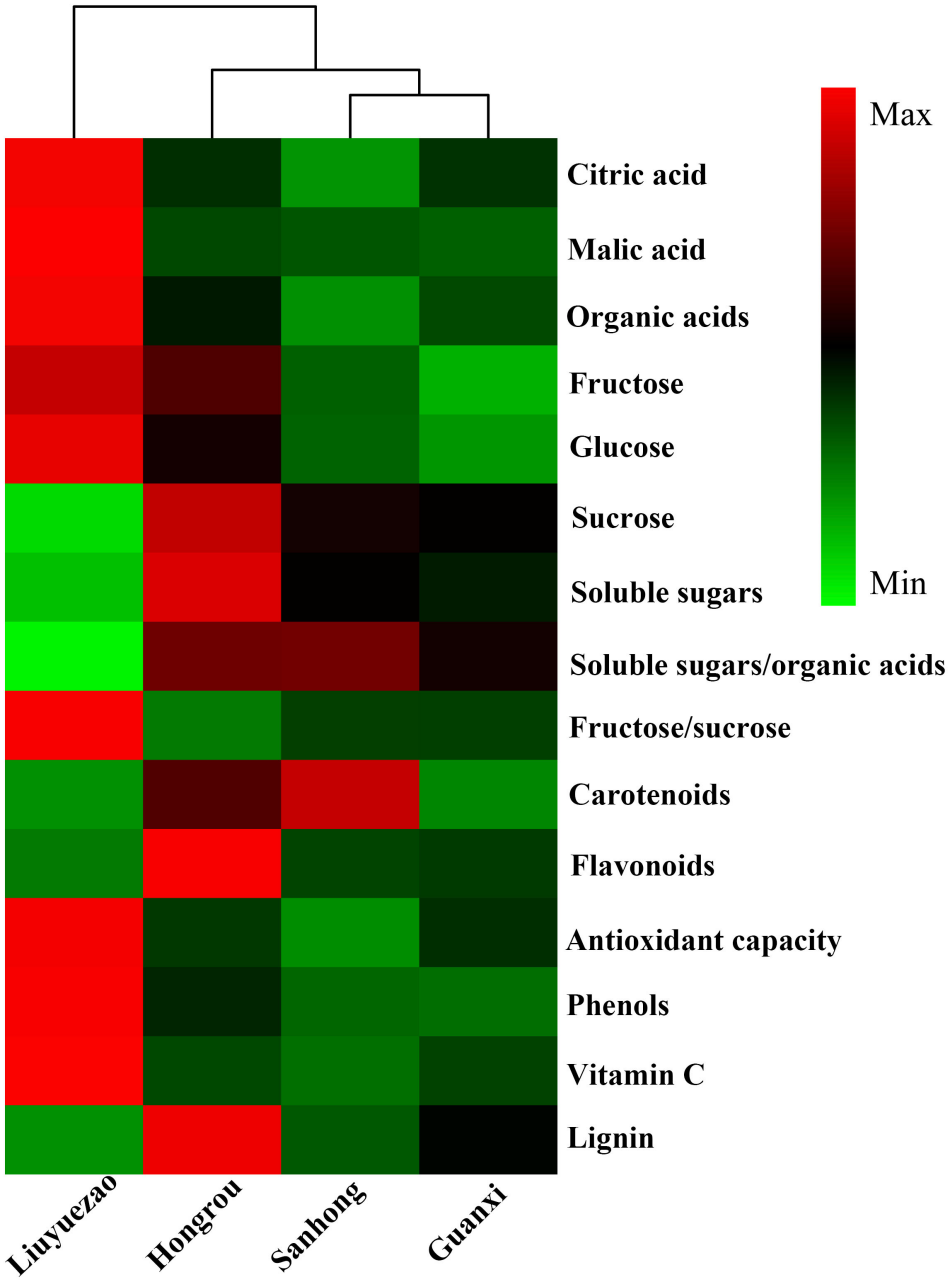
HCA of sensory and nutritional profiles in pulp samples from the four pummelo cultivars.

Principal component analysis (PCA) was employed to explore the differences in the sensory and nutritional profiles between LYZ and the other three pummelos. Component 1 and 2 (PC1 and PC2) explained 69.7% and 15.5% of the total variance, respectively (**Figure S2**). The PC1 distinguished LYZ from the other three cultivars mainly based on the vitamin C, fructose/sucrose ratio, soluble sugars/organic acids ratio, phenols, organic acids and antioxidant capacity (**Figure S2**), which indicated the significant differences in fruit quality between LYZ and the others. In addition, HCA based on sensory and nutritional quality was implemented to explore the relationship among pummelo cultivars (**Figure 2**). LYZ was divided into a separate cluster, which was consistent with the PCA (**Figure S3**). In the heatmap, it’s obvious to find the higher levels of citric acid, malic acid, organic acids, fructose, glucose, phenolics, vitamin C, fructose/sucrose ratio and antioxidation ability as well as the lower levels of soluble sugars/organic acids ratio, lignin, sucrose, soluble sugars and carotenoids in LYZ than the others.

### 3.3 Volatile profile of LYZ pulp

**Figure 3:**
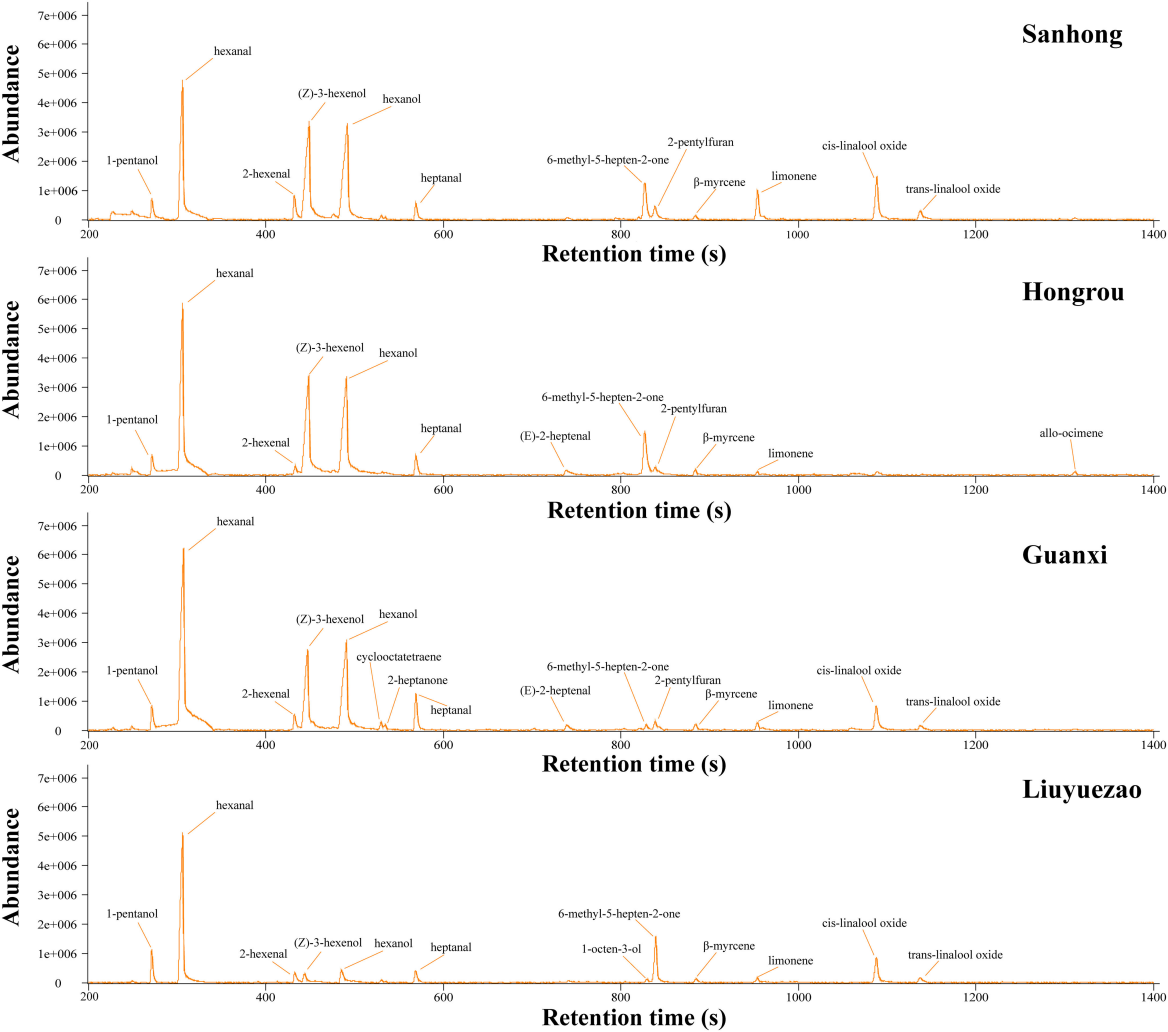
The TIC (total ion chromatogram) of pulp volatiles from the four pummelo cultivars.

**Figure 4:**
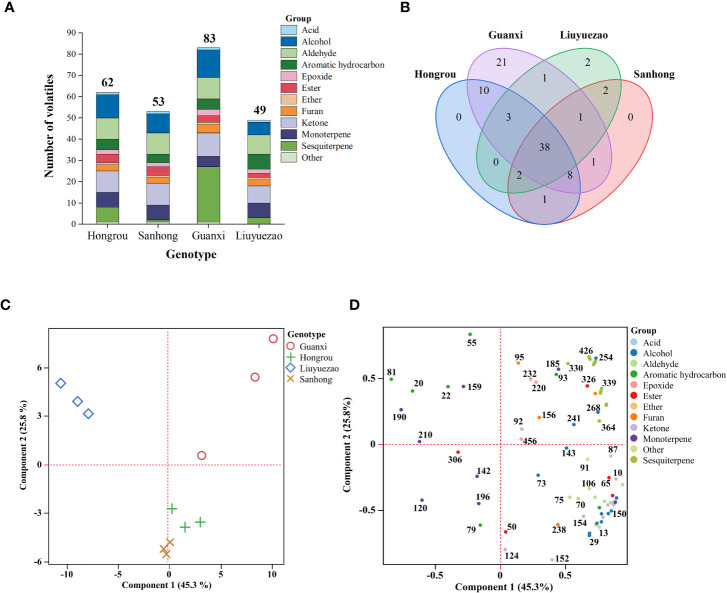
Multivariate statistical analysis of volatile profiles in pulp samples from the four pummelo cultivars. **(A)** Number of pulp volatile compounds in each chemical class. **(B)** Venn diagram analysis of pulp volatile compounds. **(C)** The score and **(D)** loading plots of principal component analysis using pulp volatiles from the four pummelo cultivars.The numbers can be found in the first column (volatile code) in **Table 3**.

The fruit aroma was an important indicator for evaluating the citrus fruit. The HS-SPME-GC-MS method was used to determine the aroma volatiles in pulp samples, and the volatile composition and content were presented from the real ionic chromatogram (TIC) (**Figure 3**). The number of pulp volatiles in LYZ was smaller when compared to HR, SH and GXB (**Figure 4A**). A total of 49 volatile compounds were identified in LYZ pulp, which included alcohols (6), aldehydes (9), aromatic hydrocarbons (7), acids (1), esters (2), epoxides (2), ethers (1), furans (3), ketones (8), monoterpenes (7) and sesquiterpenes (3) (**Figure 4A**, **Table 3**). In addition, the relative content of volatiles in LYZ pulp was 7.63, which was significantly lower than HR (28.94), SH (26.52) and GXB (23.12) (**Table 2**). Aldehydes (3.96) were the predominant chemical group of LYZ pulp, followed by monoterpenes (1.15), epoxides (1.06) and alcohols (0.94) (**Table 2**).

**Table 2:** Content of volatiles in 11 chemical classes detected in pulp among the four pummelo cultivars.

Class	Hongrou	Sanhong	Guanxi	Liuyuezao
Acid	0.016 ± 0.004 ^ab^	0.019 ± 0.002 ^ab^	0.024 ± 0.002 ^a^	0.011 ± 0.003 ^b^
Alcohol	9.645 ± 1.870 ^a^	8.600 ± 3.021 ^a^	7.258 ± 1.037 ^a^	0.939 ± 0.222 ^b^
Aldehyde	10.639 ± 2.207 ^a^	11.044 ± 1.452 ^a^	11.108 ± 1.180 ^a^	3.957 ± 0.390 ^b^
Aromatic hydrocarbon	0.247 ± 0.081 ^a^	0.147 ± 0.019 ^a^	0.401 ± 0.137 ^a^	0.165 ± 0.018 ^a^
Epoxide	2.297 ± 0.755 ^a^	0.296 ± 0.052 ^b^	1.624 ± 0.424 ^ab^	1.060 ± 0.195 ^ab^
Ester	0.020 ± 0.009 ^ab^	0.022 ± 0.007 ^a^	0.019 ± 0.001 ^ab^	0.002 ± 0.001 ^b^
Ether	0.023 ± 0.001 ^a^	0.019 ± 0.001 ^ab^	0.016 ± 0.001 ^b^	0.006 ± 0.002 ^c^
Furan	0.290 ± 0.205	0.333 ± 0.234	0.486 ± 0.045	0.099 ± 0.069
Ketone	4.917 ± 0.705 ^a^	5.425 ± 0.212 ^a^	1.150 ± 0.161 ^b^	0.237 ± 0.063 ^b^
Monoterpene	0.730 ± 0.340	0.607 ± 0.394	0.412 ± 0.001	1.149 ± 0.330
Sesquiterpene	0.091 ± 0.027 ^b^	0.004 ± 0.003 ^b^	0.379 ± 0.146 ^a^	0.004 ± 0.002 ^b^
Other	0.003 ± 0.002 ^a^	0.002 ± 0.001 ^a^	0.003 ± 0.002 ^a^	nd ^a^
Total	28.941 ± 1.399 ^a^	26.518 ± 0.887 ^a^	23.122 ± 0.919 ^b^	7.628 ± 0.129 ^c^

All values are mean of three samples per cultivar; different letters in the same rows within each tissue indicate significant differences according to Tukey’s honestly significant difference test at P < 0.05.

The content of monoterpenes was much higher in LYZ (1.15) than them in HR (0.73), SH (0.61) and GXB (0.41) even though the relative content of total volatiles in LYZ pulp was very low (**Table 2**). Monoterpenes gradually decreased while alcohols, esters and aldehydes increased during fruit ripening (Hijaz et al., 2020), which may explain the relatively high levels of monoterpenes in pulp samples from a new very early-season pummelo cultivar. 2-methylheptane and 4-methyloctane were exclusively detected in LYZ pulp (**Figure 4B** and **Table 3**), where 2-methylheptane was one of the major oil components of *Amomum Subulatum* fruits (Agnihotri et al., 2012), and 4-methyloctane was identified as one of the main volatile constitutes in mulberry (*Morus alba*) (Hwang and Kim, 2020) and black rice (*Oryza sativa*) during storage (Choi et al., 2019). Hexanal (3.51-8.51), described as green and grassy odor, was the most abundant volatile compound in pummelo pulp samples (**Table 3**), which was consistent with the results of ‘Huangjin’, ‘Sanhong’ and ‘Dongshizao’ (Zhu et al., 2020). The common aldehydes between mandarin and pummelo were hexanal and 2-hexenal (Liu et al., 2015; Yu et al., 2018). β-myrcene (0.99) was the second most abundant volatile in LYZ, which was 3.8-6.8 times than that of the other three cultivars (0.15-0.26) (**Table 3**). β-myrcene was identified as a characteristic aroma compound of Mangshanyegan (*Citrus nobilis*), which usually contributes to the formation of balsam and floral aroma (Liu et al., 2012). In addition, β-myrcene has been proven to have health-promoting properties, such as antibacterial, antioxidant, anti-inflammatory and anti-cancer (Bora et al., 2020; Galvan-Lima et al., 2021).

**Table 3:** Aroma volatiles in pulp samples among the four pummelo cultivars.

Code	Compound	Class	CAS	RI (Calculated)	RI (Published)^b^	Hongrou	Sanhong	Guanxi	Liuyuezao
7	(E)-3-penten-2-one	Ketone	625-33-2	745	735 ^c^	0.02 ± 0.01 ^zy^	0.03 ± 0.02 ^z^	0.02 ^zy^	tr ^y^
10	2-methyl-3-pentanone ^a^	Ketone	565-69-5	750	722 ^c^	tr ^zy^	tr ^z^	tr ^z^	tr ^y^
13	(E)-2-pentenal ^a^	Aldehyde	1576-87-0	753	735	0.14 ± 0.05 ^zy^	0.17 ± 0.03 ^z^	0.09 ± 0.02 ^y^	0.01 ^x^
20	2-methylheptane	Aromatic hydrocarbon	592-27-8	757	761 ^c^	nd ^z^	nd ^z^	nd ^z^	tr ^z^
22	toluene	Aromatic hydrocarbon	108-88-3	758	746 ^d^	0.02 ± 0.01 ^z^	0.02 ^z^	0.03 ± 0.01 ^z^	0.03 ^z^
27	1-pentanol ^a^	Alcohol	71-41-0	761	743	0.18 ± 0.01 ^zy^	0.19 ± 0.03 ^z^	0.13 ± 0.01 ^y^	0.05 ± 0.01 ^x^
29	(Z)-2-pentenol ^a^	Alcohol	1576-95-0	763	745	0.09 ± 0.02 ^z^	0.07 ± 0.03 ^zy^	0.04 ^yx^	tr ^x^
45	hexanal ^a^	Aldehyde	66-25-1	782	765	8.37 ± 1.95 ^z^	8.51 ± 1.38 ^z^	8.40 ± 0.97 ^z^	3.51 ± 0.33 ^y^
50	butyl acetate	Ester	123-86-4	794	778	0.01 ± 0.01 ^z^	0.01 ± 0.01 ^z^	nd ^z^	nd ^z^
55	2,4-dimethylheptane	Aromatic hydrocarbon	2213-23-2	798	820 ^d^	tr ^y^	nd ^y^	tr ^zy^	0.01 ^z^
65	ethyl 2-methylbutanoate	Ester	7452-79-1	813	856 ^f^	tr ^y^	tr ^zy^	0.01 ^z^	tr ^x^
70	(E)-2-hexenal ^a^	Aldehyde	6728-26-3	823	816	0.02 ± 0.01 ^z^	tr ^zy^	tr ^zy^	tr ^y^
73	(E)-3-hexenol ^a^	Alcohol	928-97-2	829	844	nd ^z^	0.03 ± 0.02 ^z^	0.01 ± 0.01 ^z^	nd ^z^
75	2-hexenal	Aldehyde	505-57-7	830	856 ^e^	0.37 ± 0.17 ^z^	0.21 ± 0.14 ^zy^	0.16 ± 0.06 ^zy^	0.07 ± 0.03 ^y^
78	(Z)-3-hexenol ^a^	Alcohol	928-96-1	834	818	3.61 ± 0.87 ^z^	3.25 ± 1.49 ^z^	2.30 ± 0.42 ^zy^	0.25 ± 0.10 ^y^
79	ethylbenzene ^a^	Aromatic hydrocarbon	100-41-4	834	821	0.06 ± 0.01 ^z^	0.06 ± 0.01 ^z^	0.09 ± 0.09 ^z^	0.02 ^z^
81	4-methyloctane	Aromatic hydrocarbon	2216-34-4	839	858 ^d^	nd ^y^	nd ^y^	nd ^y^	tr ^z^
84	(E)-2-hexenol ^a^	Alcohol	928-95-0	846	833	0.25 ± 0.10 ^z^	0.18 ± 0.07 ^z^	0.09 ± 0.03 ^zy^	nd ^y^
86	hexanol ^a^	Alcohol	111-27-3	850	837	5.12 ± 1.41 ^z^	4.60 ± 2.12 ^z^	4.33 ± 0.81 ^z^	0.61 ± 0.16 ^y^
87	2-methylbutanoic acid	Acid	116-53-6	854	832	0.02 ^zy^	0.02 ^zy^	0.02 ^z^	0.01 ^y^
91	2-ethylthiophene	Other	872-55-9	838	845 ^d^	tr ^z^	tr ^z^	tr ^z^	nd ^z^
92	3-heptanone	Ketone	106-35-4	863	865 ^c^	tr ^z^	tr ^z^	tr ^z^	tr ^z^
93	cyclooctatetraene	Aromatic hydrocarbon	629-20-9	866	880 ^c^	0.14 ± 0.08 ^zy^	0.05 ± 0.02 ^y^	0.25 ± 0.06 ^z^	0.09 ± 0.01 ^y^
95	2-butylfuran	Furan	4466-24-4	867	892 ^d^	nd ^z^	0.02 ± 0.03 z	0.04 ± 0.04 ^z^	tr ^z^
96	2-heptanone ^a^	Ketone	110-43-0	868	855	0.19 ± 0.04 ^z^	0.19 ± 0.04 ^z^	0.20 ± 0.02 ^z^	0.04 ± 0.01 ^y^
106	(Z)-4-heptenal	Aldehyde	6728-31-0	878	893	tr ^z^	tr ^z^	tr ^z^	nd ^z^
107	heptanal ^a^	Aldehyde	111-71-7	880	869	0.85 ± 0.19 ^y^	0.83 ± 0.08 ^y^	1.21 ± 0.11 ^z^	0.21 ± 0.06 ^x^
116	methyl hexanoate ^a^	Ester	106-70-7	907	897	tr ^zy^	tr ^zy^	tr ^z^	nd ^y^
120	α-pinene ^a^	Monoterpene	80-56-8	911	932	tr ^z^	tr ^z^	tr ^z^	tr ^z^
124	4-methyl-2-heptanone	Ketone	6137-06-0	922	936 ^c^	0.02 ^z^	0.02 ^z^	0.02 ± 0.01 ^z^	0.01 ^z^
129	4-methyl-1-hexanol	Alcohol	818-49-5	930	925 ^d^	0.05 ± 0.02 ^z^	0.05 ± 0.01 ^z^	0.06 ± 0.01 ^z^	tr ^y^
134	(E)-2-heptenal ^a^	Aldehyde	18829-55-5	945	936	0.34 ± 0.13 ^z^	0.55 ± 0.07 ^z^	0.48 ± 0.12 ^z^	0.06 ± 0.04 ^y^
142	β-pinene ^a^	Monoterpene	127-91-3	962	955	0.02 ± 0.02 ^z^	tr ^z^	nd ^z^	tr ^z^
143	(E)-2-heptenol ^a^	Alcohol	33467-76-4	962	955	tr ^z^	nd ^z^	tr ^z^	nd ^z^
144	1-heptanol ^a^	Alcohol	111-70-6	965	959	0.09 ± 0.01 ^z^	0.06 ± 0.02 ^zy^	0.06 ^y^	nd ^x^
147	1-octen-3-one	Ketone	4312-99-6	969	972	0.11 ± 0.04 ^z^	0.18 ± 0.03 ^z^	0.18 ± 0.04 ^z^	tr ^y^
150	1-octen-3-ol ^a^	Alcohol	3391-86-4	975	969	0.20 ± 0.04 ^z^	0.17 ± 0.02 ^z^	0.18 ± 0.01 ^z^	0.02 ± 0.01 ^y^
152	6-methyl-5-hepten-2-one ^a^	Ketone	110-93-0	978	971	4.14 ± 0.72 ^z^	4.55 ± 0.23 ^z^	0.42 ± 0.04 ^y^	0.13 ± 0.04 ^y^
154	3-octanone ^a^	Ketone	106-68-3	978	972	0.24 ± 0.04 ^z^	0.26 ± 0.01 ^z^	0.07 ± 0.07 ^y^	nd ^y^
156	2-pentylfuran	Furan	3777-69-3	981	984	0.27 ± 0.24 ^z^	0.30 ± 0.26 ^z^	0.43 ± 0.01 ^z^	0.09 ± 0.08 ^z^
157	2-octanone ^a^	Ketone	111-13-7	983	977	0.13 ± 0.01 ^z^	0.12 ± 0.01 ^z^	0.13 ^z^	0.04 ± 0.02 ^y^
159	β-myrcene ^a^	Monoterpene	123-35-3	983	976	0.26 ± 0.08 ^y^	0.15 ± 0.13 ^y^	0.15 ± 0.03 ^y^	0.99 ± 0.33 ^z^
172	octanal ^a^	Aldehyde	124-13-0	998	994	0.25 ± 0.06 ^z^	0.26 ± 0.03 ^z^	0.26 ± 0.03 ^z^	0.08 ± 0.02 ^y^
185	p-cymene	Monoterpene	99-87-6	1014	1020	tr ^z^	nd ^z^	tr ^z^	tr ^z^
190	m-cymene	Monoterpene	535-77-3	1019	1045 ^f^	nd ^y^	tr ^zy^	nd ^y^	tr ^z^
196	limonene ^a^	Monoterpene	138-86-3	1024	1021	0.36 ± 0.35 ^z^	0.36 ± 0.36 ^z^	0.12 ± 0.06 ^z^	0.15 ± 0.07 ^z^
197	eucalyptol	Ether	470-82-6	1026	1026	0.02 ^z^	0.02 ^zy^	0.02 ^y^	tr ^x^
209	2-nonanone ^a^	Ketone	821-55-6	1048	1087	0.06 ± 0.02 ^z^	0.07 ± 0.01 ^z^	0.08 ± 0.01 ^z^	nd ^y^
210	cis-β-ocimene	Monoterpene	3338-55-4	1048	1032	tr ^z^	tr ^z^	nd ^z^	tr ^z^
214	(E)-2-octenal ^a^	Aldehyde	2548-87-0	1064	1063	0.25 ± 0.13 ^zy^	0.44 ± 0.07 ^z^	0.45 ± 0.12 ^z^	0.02 ± 0.03 ^y^
220	cis-linalool oxide	Epoxide	5989-33-3	1074	1067	1.75 ± 0.69 ^z^	0.24 ± 0.05 ^y^	1.25 ± 0.39 ^zy^	0.81 ± 0.19 ^zy^
232	trans-linalool oxide	Epoxide	34995-77-2	1093	1084	0.54 ± 0.23 ^z^	0.06 ± 0.01 ^y^	0.37 ± 0.13 ^zy^	0.25 ± 0.05 ^zy^
233	2-hexylfuran	Furan	3777-70-6	1097	1094 ^c^	tr ^z^	tr ^z^	0.01 ^z^	tr ^z^
238	perillene	Furan	539-52-6	1106	1102	0.01 ^z^	0.02 ± 0.01 ^z^	tr ^y^	tr ^y^
241	linalool ^a^	Alcohol	78-70-6	1111	1112	0.01 ± 0.01 ^z^	nd ^z^	tr ^z^	nd ^z^
243	β-thujone	Ketone	471-15-8	1115	1101	nd ^z^	nd ^z^	0.03 ± 0.03 ^z^	nd ^z^
246	nonanal ^a^	Aldehyde	124-19-6	1117	1117	0.04 ± 0.02 ^zy^	0.05 ± 0.01 ^z^	0.05 ± 0.01 ^z^	tr ^y^
254	trans-p-mentha-2,8-dien-1-ol	Alcohol	7212-40-0	1137	1119	nd ^y^	nd ^y^	tr ^z^	nd ^y^
257	allo-ocimene	Monoterpene	7216-56-0	1141	1128	0.09 ± 0.04 ^z^	0.10 ± 0.03 ^z^	0.14 ± 0.03 ^z^	nd ^y^
268	trans-p-2-menthen-1-ol	Alcohol	29803-81-4	1158	1136	0.03 ± 0.03 ^zy^	nd ^y^	0.05 ± 0.01 ^z^	nd ^y^
306	ethyl 3-hydroxyhexanoate	Ester	2305-25-1	1127	1128 ^e^	nd ^z^	tr ^z^	nd ^z^	tr ^z^
308	1,3-ditert-butylbenzene	Aromatic hydrocarbon	1014-60-4	1267	1245 ^c^	0.02 ± 0.01 ^z^	0.02 ^z^	0.02 ^z^	tr ^y^
326	(3Z)-hexenyl tiglate	Ester	84060-80-0	1346	1319	tr ^z^	nd ^z^	tr ^z^	nd ^z^
328	elemene isomer	Sesquiterpene		1351	–	nd ^y^	nd ^y^	tr ^z^	nd ^y^
330	δ-elemene ^a^	Sesquiterpene	20307-84-0	1355	1369	0.01 ^y^	nd ^y^	0.06 ± 0.03 ^z^	tr ^y^
334	α-cubebene	Sesquiterpene	17699-14-8	1366	1345	nd ^y^	nd ^y^	tr ^z^	nd ^y^
341	α-ylangene	Sesquiterpene	14912-44-8	1386	1373	nd ^y^	nd ^y^	tr ^z^	nd ^y^
346	(-)-β-bourbonene	Sesquiterpene	5208-59-3	1400	1387	0.01 ^zy^	nd ^y^	0.06 ± 0.04 ^z^	nd ^y^
347	RI1400	Sesquiterpene		1400	–	nd ^y^	nd ^y^	tr ^z^	nd ^y^
352	β-elemene ^a^	Sesquiterpene	515-13-9	1407	1423	tr ^y^	nd ^y^	0.02 ± 0.01 ^z^	nd ^y^
355	RI1411	Sesquiterpene		1411	–	nd ^z^	nd ^z^	tr ^z^	nd ^z^
360	α-gurjunene	Sesquiterpene	489-40-7	1422	1409	nd ^z^	nd ^z^	tr ^z^	nd ^z^
364	(Z)-caryophyllene	Sesquiterpene	118-65-0	1434	1408	0.07 ± 0.02 ^y^	tr ^y^	0.20 ± 0.08 ^z^	tr ^y^
370	β-cubebene	Sesquiterpene	13744-15-5	1443	1463 ^f^	nd ^z^	nd ^z^	tr ^z^	nd ^z^
371	γ-elemene	Sesquiterpene	29873-99-2	1445	1434	nd ^y^	nd ^y^	0.01 ± 0.01 ^z^	nd ^y^
378	(Z)-β-farnesene	Sesquiterpene	28973-97-9	1456	1440	nd ^z^	nd ^z^	tr ^z^	nd ^z^
382	cadina-3,5-diene	Sesquiterpene	267665-20-3	1458	1448	nd ^z^	nd ^z^	tr ^z^	nd ^z^
383	cis-muurola-3,5-diene	Sesquiterpene	157374-44-2	1461	1448	nd ^z^	nd ^z^	tr ^z^	nd ^z^
385	α-humulene	Sesquiterpene	6753-98-6	1466	1452	tr ^y^	nd ^y^	0.01 ± 0.01 ^z^	nd ^y^
390	cis-muurola-4(15),5-diene	Sesquiterpene	157477-72-0	1473	1465	nd ^z^	nd ^z^	tr ^z^	nd ^z^
391	RI1478	Sesquiterpene		1478	–	nd ^z^	nd ^z^	tr ^z^	nd ^z^
399	γ-muurolene	Sesquiterpene	30021-74-0	1487	1478	tr ^y^	nd ^y^	0.03 ± 0.01 ^z^	nd ^y^
402	germacrene D	Sesquiterpene	23986-74-5	1491	1484	0.02 ± 0.01 ^y^	nd ^y^	0.15 ± 0.09 ^z^	nd ^y^
406	δ-selinene	Sesquiterpene	28624-28-4	1497	1492	nd ^z^	nd ^z^	tr ^z^	nd ^z^
410	valencene	Sesquiterpene	4630-07-3	1502	1496	nd ^y^	nd ^y^	tr ^z^	nd ^y^
414	α-muurolene	Sesquiterpene	10208-80-7	1508	1500	nd ^z^	nd ^z^	tr ^z^	nd ^z^
420	γ-cadinene	Sesquiterpene	39029-41-9	1520	1513	nd ^z^	nd ^z^	0.01 ± 0.01 ^z^	nd ^z^
426	δ-cadinene ^a^	Sesquiterpene	483-76-1	1526	1550	nd ^y^	nd ^y^	0.02 ± 0.02 ^z^	tr ^y^
444	germacrene B	Sesquiterpene	15423-57-1	1559	1559	nd ^y^	nd ^y^	tr ^z^	nd ^y^
456	caryophyllene oxide	Epoxide	1139-30-6	1594	1582	nd ^z^	nd ^z^	tr ^z^	nd ^z^

Data are normalized to the internal standard peak area. All values are means of three biological replicates per genotype. Retention indices were calculated from a mixture of C8-C20. RI: retention index; tr: peak was detected but the value less than 0.0095; nd: not detected. ^a^ Identified by authentic volatile compound standards. ^b^ Published retention indices on DB-5 column reported by (Adams, 2007) unless mentioned otherwise. ^c^ Published retention indices on DB-5 column reported on PubChem (Kim et al., 2021). ^d^ Published retention indices on DB-5 column reported on NIST Chemistry WebBook (Linstrom and Mallard, 2022). ^e^ Published retention indices on DB-5 column reported on Flavornet and Human Odor Space (Acree and Arn, 2004). ^f^ Published retention indices on DB-5 column reported by (Miyazaki et al., 2011). ^z-x^ Different letters in the same rows indicate significant differences according to Tukey’s honestly significant difference test at P < 0.05.

**Figure 5:**
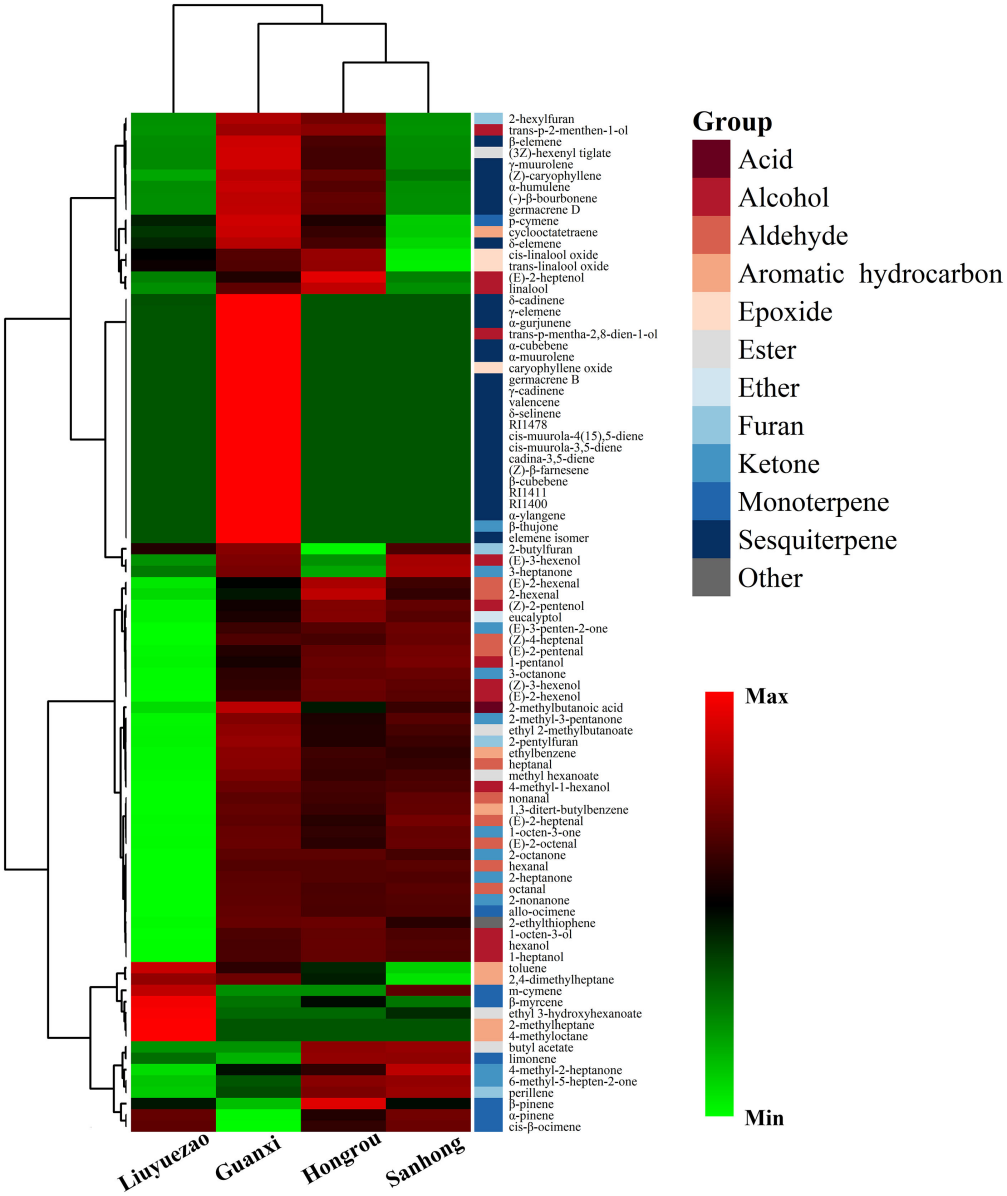
HCA of volatile profiles in pulp samples from the four pummelo cultivars.

PCA and HCA directly showed the relationship between the volatiles and samples, and were becoming more and more extensive in the aroma volatile study (Wei et al., 2018). PC1 and PC2 accounted for 45.3% and 25.8% of the total variance, respectively (**Figure 4C**). PC1 separated LYZ from the other pummelos mainly based on several volatiles, including heptanal (107, peak code as described in **Table 3**), 4-methyl-1-hexanol (129), 2-methyl-3-pentanone (10), allo-ocimene (257), 1-octen-3-one (147), 2-heptanone (96), 2-nonanone (209), octanal (172), methyl hexanoate (116) and hexanal (45) (**Figure 4D** and **Table S1**). In addition, 6-methylhept-5-en-2-one (152), 2,4-dimethylheptane (55), 4-methyl-2-heptanone (124), 1-pentanol (27), (Z)-2-pentenol (29), butyl acetate (50), δ-cadinene (426) and trans-p-mentha-2,8-dien-1-ol (254) contributed to separate the samples across PC2 (**Figure 4D** and **Table S1**). HCA based on the volatile profiles distinguished LYZ from HR, SH and GXB, which validated the PCA results (**Figure 5**). Several aromatic hydrocarbons and monoterpenes including toluene (22), 2,4-dimethylheptane (55), m-cymene (190), β-myrcene (159), ethyl 3-hydroxyhexanoate (306), 2-methylheptane (20) and 4-methyloctane (81) were abundant in LYZ pulp (**Figure 5** and **Table 3**). Toluene was presented at a high level in citrus peel (Ligor and Buszewski, 2003), and 2,4-dimethylheptane could be used as an indicator of the geographical origin of ‘Ntopia’ olive oil among the four main Ionian islands (Eriotou et al., 2021). m-Cymene was previously reported in ‘Valencia’ orange leaf oil (Moshonas and Shaw, 1986), and showed certain degrees of insecticidal and repellent activities to *Tribolium castaneum* and *Liposcelis bostrychophila* (Feng et al., 2021). Ethyl 3-hydroxyhexanoate is an odor -active compound with a fruity and sweet odor in orange juice (Hinterholzer and Schieberle, 1998; Buettner and Schieberle, 2001). In conclusion, the low level of total fruit volatiles and unique volatile composition in LYZ generated its moderate aroma of pummelo note.

It was reported that adding acid generally increased aroma and sourness and reduced sweetness, fruity and bitterness, and adding sugar generally reduced the perception of sour, bitter and citrus flavors (Baldwin et al., 2008). Here, the pairwise correlation analysis between fruit qualities and selected volatile groups was performed. Alcohols and aldehydes both had a significant positive correlation with soluble sugars (0.750, 0.646) and soluble sugars/organic acids ratio (0.833, 0.828), and had a significant negative correlation with organic acids (-0.774, -0.828), phenols (-0.772, -0.894), vitamin C (-0.850, -0.865), antioxidants (-0.792, -0.879) and fructose/sucrose ratio (-0.878, -0.863) (**Figure S3**). Aldehydes and alcohols were positively correlated with sweetness and negatively correlated with acidity (Hijaz et al., 2020). In addition, alcohols and aldehydes were negatively correlated with antioxidant capacity. Ketones were positively correlated with soluble sugars (0.735) and carotenoids (0.966) but negatively correlated with fructose/sucrose ratio (-0.710) and antioxidant capacity (0.771) (**Figure S3**). Monoterpenes showed positive correlations with organic acids (0.606), phenolics (0.625) and vitamin C (0.584) (**Figure S3**). It was reported that monoterpenes were positively correlated with bitterness and orange taste but negatively correlated with sweetness (Hijaz et al., 2020), and monoterpenes were also positively correlated with antioxidant capacity in sweet oranges (Sanchez-Martinez et al., 2021).

Aldehydes were positively correlated with ketones (0.653) but negatively correlated with monoterpenes (-0.598) (**Figure S3**). In mandarin fruits, aldehydes were positively correlated with esters and ketones (Yu et al., 2018). Moreover, we found the correlation coefficients between some typical volatile compounds in pummelo pulp. Cis-linalool oxide (220) and trans-linalool oxide (232) had an extremely high correlation coefficient (0.999), and were also closely clustered in the HCA (**Figure 5**). (Z)-3-hexenol (78) and hexanol (86) were distributed under the same major branch and had a high correlation coefficient (0.969) (**Figure 5**). Hexanal (45) was in the same small cluster with (E)-2-heptenal (134) and they had a correlation coefficient of 0.914 (**Figure 5**). Hexanal also had a positive correlation with heptanal (107) (0.899) and they were in the same branch in HCA (**Figure 5**).

## 4 Conclusion

This study introduced the fruit qualities and aroma volatiles from the new very early-season pummelo cultivar LYZ. Compared to the other major pummelo cultivars, LYZ significantly differed in sensory and nutritional qualities. LYZ had a relatively high fructose content and the fructose/sucrose ratio of LYZ was close to 1. LYZ fruit was described as sweet and mildly sour taste, which was mainly caused by the higher content of organic acids and fructose and the lower content of sucrose and lignin. In addition, LYZ pulp was rich in phenols and vitamin C, and had a strong antioxidant capacity. The low level of total fruit volatiles and unique volatile composition in LYZ contributed to its moderate aroma of pummelo note. Moreover, the PCA and HCA based on fruit qualities and volatile profiles differentiated LYZ from the other three pummelos. Therefore, LYZ is a new option of pummelo available with extremely early maturity and unique fruit flavor.

## Data availability statement

The original contributions presented in the study are included in the article/**Supplementary Material**. Further inquiries can be directed to the corresponding author.

## Author contributions

YY designed and coordinated the study. TP, LK, XZ, YW, JZ and ZF collected the fruit samples and performed the experiments. LK, JZ, HP and WS analyzed the data. LK and YY wrote the manuscript with input from all authors. All authors contributed to the article and approved the submitted version.
