# Supplementary material for: Fruit quality and volatile constituents of a new very early-ripening pummelo (Citrus maxima) cultivar ‘Liuyuezao’
Source: Front Plant Sci. 2023 Jan 9;13:1089009. doi: 10.3389/fpls.2022.1089009 (PMC9868557; doi:10.3389/fpls.2022.1089009)
Supplement: Supplementary file 1 [file DataSheet_1.pdf]

Supplementary Figures and Tables

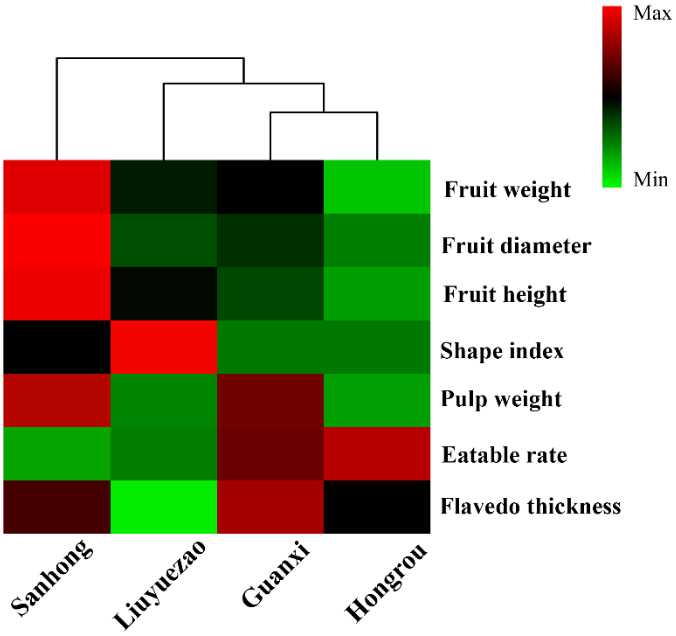

Figure S1. HCA of fruit characteristics of the four pummelo cultivars.

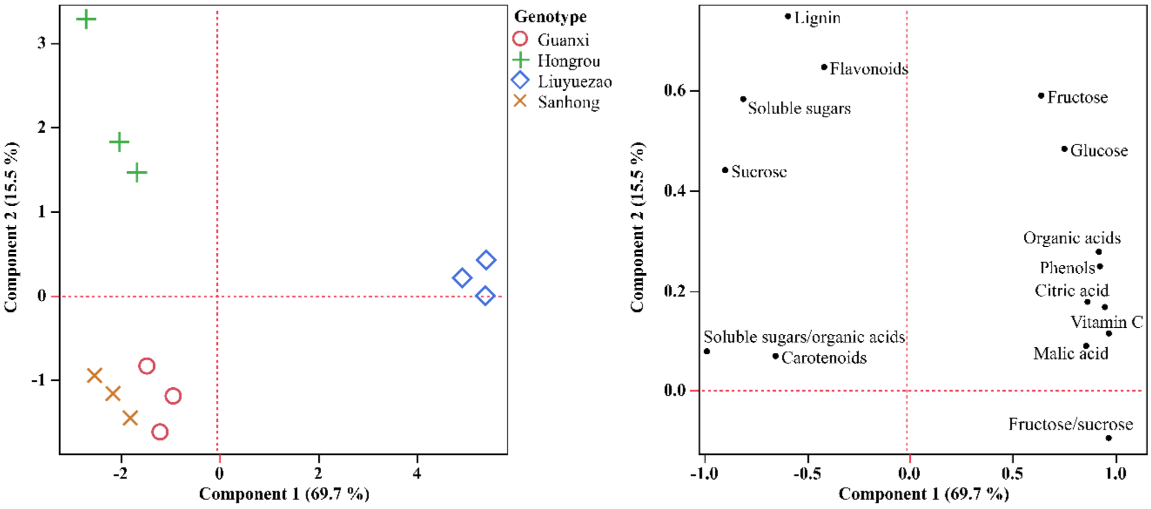

Figure S2. Principal component analysis of sensory and nutritional profiles in pulp samples from the four pummelo cultivars. (A) The score and (B) loading plots of principal component analysis.

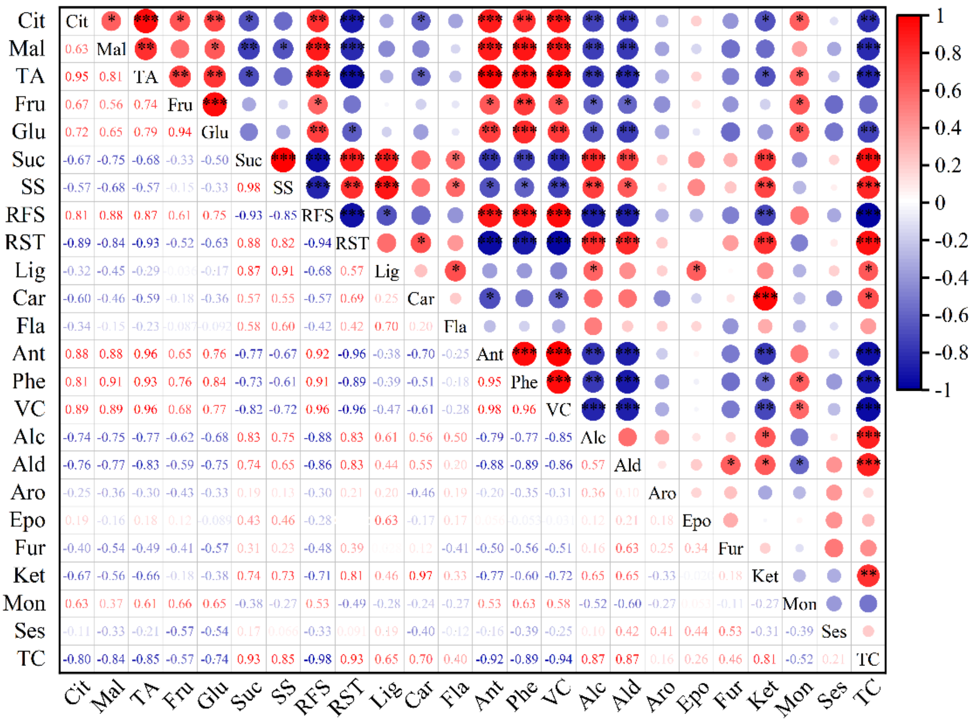

Figure S3. Correlation analysis heatmap of sensory and nutritional traits. The sensory and nutritional traits included citric acid (Cit), malic acid (Mal), organic acids (TA), fructose (Fru), glucose (Glu), sucrose (Suc), soluble sugars (SS), fructose/sucrose (RFS), soluble sugars/organic acids (RST), lignin (Lig), carotenoids (Car), flavonoids (Fla), antioxidant capacity (Ant), phenols (Phe), vitamin C (VC) and volatile alcohols (Alc), aldehydes (Ald), aromatic hydrocarbons (Aro), epoxides (Epo), furans (Fur), ketones (Ket), monoterpenes (Mon), sesquiterpenes (Ses) and total volatile content (TC). * *P* < 0.05, ** *P* < 0.01, *** *P* < 0.01.

Table S1 Compounds with high contribution to the PC1 and PC2 in Figure 4C

**Code**	**Compound**	**Class**	**PC1**	**PC2**
107	heptanal	Aldehyde	**0.88**	0.10
129	4-methyl-1-hexanol	Alcohol	**0.80**	0.17
10	2-methyl-3-pentanone	Ketone	**0.79**	0.07
257	allo-ocimene	Monoterpene	**0.78**	0.20
147	1-octen-3-one	Ketone	**0.78**	0.19
96	2-heptanone	Ketone	**0.76**	0.21
209	2-nonanone	Ketone	**0.76**	0.21
172	octanal	Aldehyde	**0.76**	0.20
116	methyl hexanoate	Ester	**0.75**	0.15
45	hexanal	Aldehyde	**0.74**	0.20
150	1-octen-3-ol	Alcohol	**0.73**	0.26
87	2-methylbutanoic acid	Acid	**0.72**	0.01
134	(E)-2-heptenal	Aldehyde	**0.72**	0.20
65	ethyl 2-methylbutanoate	Ester	**0.70**	0.07
81	4-methyloctane	Aromatic hydrocarbon	**0.69**	0.24
152	6-methyl-5-hepten-2-one	Ketone	0.16	**0.77**
55	2,4-dimethylheptane	Aromatic hydrocarbon	0.05	**0.69**
124	4-methylheptan-2-one	Ketone	0.00	**0.64**
27	1-pentanol	Alcohol	0.47	**0.48**
29	(Z)-2-pentenol	Alcohol	0.47	**0.46**
50	butyl acetate	Ester	0.00	**0.45**
426	δ-cadinene	Sesquiterpene	0.47	**0.44**
254	trans-p-mentha-2,8-dien-1-ol	Alcohol	0.54	**0.42**
378	(Z)-β-farnesene	Sesquiterpene	0.47	**0.42**
383	cis-muurola-3,5-diene	Sesquiterpene	0.47	**0.42**
406	δ-selinene	Sesquiterpene	0.47	**0.42**
360	α-gurjunene	Sesquiterpene	0.47	**0.42**
382	cadina-3,5-diene	Sesquiterpene	0.48	**0.42**
391	RI1478	Sesquiterpene	0.48	**0.42**
370	β-cubebene	Sesquiterpene	0.48	**0.42**
